# Evaluating infection prevention and control structure of Indonesian COVID-19 referral hospitals

**DOI:** 10.4102/jamba.v15i1.1466

**Published:** 2023-07-25

**Authors:** Ekorini Listiowati, Mohammad A. Samsudin, Yuanita Wulandari, Cintyanna Taritasari, Mundakir Mundakir, Mochamad I. Nurmansyah

**Affiliations:** 1 Department of Public Health, Faculty of Medicine and Health Sciences, Universitas Muhammadiyah Yogyakarta, Yogyakarta, Indonesia; 2 Department of Management, Faculty of Economics, Social and Humanities, Universitas Aisyiyah Yogyakarta, Indonesia; 3 Department of Nursing, Faculty of Health Sciences, Universitas Muhammadiyah Surabaya, Surabaya, Indonesia; 4 Health Division, Central Board of Muhammadiyah, Jakarta, Indonesia; 5 Department of Public Health, Faculty of Health Sciences, Universitas Islam Negeri Syarif Hidayatullah, Jakarta, Indonesia

**Keywords:** communicable diseases, prevention control, healthcare, developing countries, COVID-19

## Abstract

Due to the emergence of COVID-19, hospitals are required to increase vigilance in providing care. However, their readiness for infection prevention and control (IPC) as a referral hospital in providing COVID-19 services has not been determined. This study aims to evaluate the IPC structure of 30 private non-profit Indonesian referral hospitals for COVID-19 based on the World Health Organization Infection Prevention and Control Assessment Framework (WHO IPCAF). A descriptive cross-sectional quantitative study was used, where 30 hospitals as the COVID-19 referral hospital were selected. The data collection was conducted by an online survey using the IPCAF questionnaire created by the WHO and was analysed with descriptive analysis. The majority of the hospitals’ IPC level is at an advanced level (73.3%). All type B hospitals have an advanced IPC level, while only 64.7% of type C and 71.4% of type D have an advanced level. The highest average IPC score is on the IPC guidelines component (94.0), while the lowest value of 71.9 is on the Surveillance of HAIs component. In the minimum scores, there were hospitals with the lowest scores in HAI Surveillance and Multimodal strategies, namely 20.0 and 25.0, respectively. Preparing human resource capacities, establishing functional programmes, developing and implementing IPC guidelines, and providing adequate supplies are needed to improve hospital IPC structures.

**Contribution:**

This study demonstrates the necessity to improve hospital IPC structures to increase the resilience of health services to natural hazards and public health emergencies.

## Introduction

Healthcare-associated infections (HAIs) are among the common causes of preventable harm and a major public health problem (Zimlichman et al. 2013). It is estimated that 1 in every 10 patients is affected by HAIs worldwide, while in acute care hospitals, out of every 100 patients, 7 in developed and 15 in developing countries will acquire at least one HAI (World Health Organization 2016). The impact of HAI implies prolonged hospital stay, long-term disability, increased resistance of microorganisms to antimicrobials, massive additional financial burden, high costs for patients and their families, and a high death rate (World Health Organization 2009b). Moreover, investigations have revealed that the prevalence of HAIs in developing countries is twice as severe compared to developed countries (Allegranzi et al. 2011). However, accurate estimates of the burden of HAI in developing nations cannot be made due to a lack of surveillance programmes and data at national level (Bammigatti et al. 2017).

Recently, HAI rates increased due to COVID-19 pandemic, particularly in resource-limited countries (Lastinger et al. 2022; Rosenthal et al. 2022). Moreover, Indonesia is one of the countries with the highest number of COVID-19 cases in the world and became an epicentre of the pandemic in 2021 (Gamalliel, Saminarsih & Taher 2021). The Indonesian government has appointed hundreds of public and private hospitals as referral hospitals for handling COVID-19. Because an increase in HAIs can occur during a pandemic, health services need to implement infection prevention and control (IPC), as a practical measure based on scientific evidence which protects health workers, patients, and visitors to healthcare facilities (Tomczyk et al. 2022). Several studies demonstrated an increase in HAIs during the COVID-19 pandemic (Deiana et al. 2022; Halverson et al. 2022; Lastinger et al. 2022). According to various studies, up to 41% of hospitalised patients with confirmed COVID-19 were infected in healthcare settings (Abbas et al. 2021). The prevalence of infection varied from 0.3% to 43.3% among health workers (Chou et al. 2020). Another study revealed that as of 08 May 2020, 152 888 healthcare workers were reported to be infected with COVID-19 (Bandyopadhyay et al. 2020).

In the context of the disaster, the pandemic of COVID-19 has been categorised as a natural hazard (Kelman 2020). There is a correlation between the classification and the type of disaster, vulnerability, and exposure (Seddighi 2020). In addition, healthcare workers have emerged as a vulnerable population group during COVID-19, and securing the supply chains of personal protective equipment (PPE) has been identified as a crucial issue for protecting healthcare workers and preventing health system overload (Smith 2020). In addition, the Indonesian government has declared the COVID-19 pandemic a national disaster through a Presidential Decree (Cabinet Secretary of the Republic Indonesia 2020).

Infection prevention and control is one of the important programmes to maintain the safety of patients and health workers during health services in hospitals. This is because the prevention of HAIs can reduce morbidity, mortality, and healthcare costs (Haque et al. 2018). Planning and implementing the right IPC programme can also reduce HAIs and save hospital expenses (Godfrey & Schouten 2014). There is a need to carry out IPC by referring to the established guidelines. In Indonesia, hospitals refer to the guidelines on core components of IPC programmes developed by the World Health Organization (WHO) to create and strengthen the system. The achievement of each IPC component can be measured using the Infection Prevention and Control Assessment Framework (IPCAF) to measure the level of the relevant hospital. However, there is limited information on the IPC structures of Indonesian hospitals, particularly referral hospitals for COVID-19.

Several IPC measurement-related studies were conducted prior to the pandemic (Johannes et al. 2019; Savul et al. 2020; Tomczyk et al. 2020). As the current COVID-19 pandemic continues to emphasise the significance of IPC and its sound establishment in healthcare facilities, a number of studies have been conducted to measure IPCAF during the pandemic (Harun et al. 2022; Jeong et al. 2022; Kamara et al. 2022). Unfortunately, the topic remains understudied in Indonesia, and only few studies measuring IPCAF during the pandemic have been identified (Supriadi et al. 2023). However, the average prevalence of nosocomial infections is approximately 9.1%, according to a study (Haryanto, Keperawatan & Rajawali 2022). Therefore, this study aims to evaluate the IPC structure of 30 private non-profit Indonesian referral hospitals for COVID-19 based on the World Health Organization Infection Prevention and Control Assessment Framework (WHO IPCAF). This result is expected to be baseline data for hospital managers and clinicians to improve hospital IPC (Tomczyk et al. 2020).

## Methods

A cross-sectional survey was carried out in October 2020 at 30 private non-profit COVID-19 referral hospitals owned by an Indonesian socioreligious organisation called Muhammadiyah. Muhammadiyah is one of the organisations that has become one of the most agile promoters of health and hospital-based emergency responses (Ichsan 2022). Muhammadiyah hospitals designated by the local government as a COVID-19 referral facility have been selected in this study. In addition, we take into account the high number of COVID-19 cases in Indonesia’s various regions. Therefore, 30 Muhammadiyah hospitals in Indonesia met these criteria. The selected hospitals are located in the central and western provinces of Jakarta, Yogyakarta, Central Java, East Java, South Sumatra, South Kalimantan, and Central Kalimantan. The selected hospitals comprised 6 type B, 17 type C, and 7 type D hospitals. According to Indonesian Ministry of Health, a hospital in Indonesia is categorised based on the number of inpatient beds, where the types B, C, and D have a minimum of 200, 100, and 50 beds, respectively.

The data were collected by using a self-report online questionnaire. The hospital was sent an online questionnaire by the researcher. The hospital’s IPC committee or team completed and returned the informed consent and questionnaire to the researcher. Institutional approval from hospital managements has been obtained prior to study. In the initial informed consent form, the participants received a description of the objectives and rationale behind the study. The participants who agreed to fill out the form gave their informed consent for the research, and followed the Declaration of Helsinki principles.

The WHO IPCAF is an instrument that has been widely used by researchers in various parts of the world, both developed and developing countries, to measure IPC levels in hospitals (Johannes et al. 2019; Oppong et al. 2020; Savul et al. 2020). In this study, the instrument was used to determine the IPC structures of the selected hospitals. The WHO IPCAF measures eight IPC core components, namely the IPC programme (CC1) (10 questions), IPC guidelines (CC2) (8 questions), IPC education and training (CC3) (10 questions), HAIs surveillance (CC4) (15 questions), multimodal strategies for implementation of IPC interventions (CC5) (5 questions), monitoring/auditing of IPC practices and feedback (CC6) (8 questions), workload, staffing, and bed occupancy (CC7) (8 questions), and built environment, materials, and equipment for IPC at the facility level (CC8) (17 questions). Each question has a response between ‘Yes’ and ‘No’ and a ‘Yes’ response with an explanation. According to the instructions, each answer has a score ranging from 0 to 15 (World Health Organization 2018).

The score ranges from 0 to 100 per core component. The final IPCAF score is the calculation of the total score from the eight core components with a maximum value of 800. Based on this score, the IPC level is divided into four levels, namely (1) inadequate (0–200 points), inadequate IPC implementation with a significant increase is required, (2) basic (201–400 points), some aspects of functional IPC without adequate implementation with additional enhancements required, (3) intermediate (401–600 points), most aspects of the IPC are properly implemented, and (4) advanced (601–800 points), the IPC core components are implemented according to WHO recommendations and facility requirements (World Health Organization 2018). Subsequently, descriptive analysis using Microsoft Excel was performed to show the frequency, percentages, mean, minimum, and maximum values of each variable.

### Ethical considerations

Ethical clearance to conduct this study was obtained from the PKU Muhammadiyah Gamping Hospital, Research Ethic Commission (No. 0003A/KEP-PKU/VIII/2020).

## Results

A total of 30 non-profit hospitals were identified in various regions in Indonesia, where 11 and 12 of the hospitals came from East Java and Central Java, respectively. Each of the two hospitals is from Jakarta and Yogyakarta, while the rest are from Central Kalimantan, Lampung, and South Sumatra. Furthermore, 6 hospitals come from type B, 17 are type C, and 7 are type D. From Table 1, the majority of the hospitals’ IPC level is at an advanced level (73.3%) based on the class of hospital. All type B hospitals were advanced IPC level, while only 64.7% of type C and 71.4% of type D have an advanced level (Table 1).

**TABLE 1:** Infection prevention and control level based on the class of hospitals.

Hospital class	Number of hospitals		IPC level			
			Intermediate		Advance	
	*n*	%	*n*	%	*n*	%
B	6	20.0	0	0	6	100.0
C	17	56.7	6	35.3	11	64.7
D	7	23.3	2	28.6	5	71.4

**Total**	**30**	**100**	**8**	**26.7**	**22**	**73.3**

IPC, infection prevention and control.

Table 2 shows the characteristics of IPC core components based on hospital class. The results showed that type B hospitals had the highest scores on all IPC components, except for CC8 where type C and D hospitals had the highest. Type D hospitals have the lowest IPC scores on the components of surveillance and strategies for implementation. Meanwhile, type C hospitals also have the lowest score on the CC4 component.

**TABLE 2:** Characteristics of infection prevention and control core components based on hospital class.

Core components	All	Type D	Type C	Type B
IPC programmes (CC1)	88.0	83.2	88.4	92.5
IPC guidelines (CC2)	94.0	96.8	91.8	97.1
IPC education and training (CC3)	83.2	84.3	80.6	89.2
HAI surveillance (CC4)	71.9	71.4	66.0	89.2
Multimodal strategies for implementation of IPC (CC5)	72.7	74.3	69.4	80.0
Monitoring of IPC practices (CC6)	78.5	77.3	77.1	83.8
Workload, staffing and bed occupancy (CC7)	77.0	82.1	72.6	83.3
Environment, materials, and equipment for IPC (CC8)	85.4	88.6	87.8	75.0
Average all components	81.7	82.3	79.2	88.2

HAI, healthcare-associated infection; IPC, infection prevention and control.

The highest average IPC score is on the CC2 component (94.0), while the lowest is on CC4, which is 71.9. In the minimum and maximum scores, there were hospitals with the lowest scores in the CC4 and CC5 of 20.0 and 25.0, respectively, as shown in Table 2. This indicates that surveillance of HAIs is important to inform and guide IPC strategies as well as IPC activities using multimodal strategies. This was also implemented to improve practices and reduce HAIs and Antimicrobial Resistance (AMR). Moreover, some hospitals have IPC education and training (CC3) as well as workload, staffing, and bed occupancy (CC7), which are categorised in the basic category (Table 3).

**TABLE 3:** Infection prevention and control core components score and average.

IPC core component	Mean	Minimum	Maximum
IPC programme (CC1)	88.0	67.5	100.0
IPC guidelines (CC2)	94.0	72.5	100.0
IPC education and training (CC3)	83.2	45.0	100.0
HAI surveillance (CC4)	71.9	25.0	100.0
Multimodal strategies for implementation IPC interventions (CC5)	72.7	20.0	100.0
Monitoring/audit of IPC practices and feedback (CC6)	78.5	53.5	90.0
Workload, staffing, and bed occupancy (CC7)	77.0	45.0	100.0
Built environment, materials, and equipment for IPC at the facility level (CC8)	85.4	77.5	100.0

IPC, infection prevention and control; HAI, healthcare-associated infection.

Notes: Maximum score for each core component was 100. Component levels: 0% – 25% = inadequate; 25.1% – 50% = basic; 50.1% – 75% = intermediate; 75.1% – 100% = advanced.

## Discussion

The study discovered that majority of hospitals (73.3%) have an advanced level of IPC level. This value is lower compared to an investigation conducted in Eastern China, where it was reported that 82.9% of hospitals were at the advanced IPC level (Ni et al. 2022). A previous study in Germany also stated that approximately 85% of hospitals were classified into the advanced IPC level (Johannes et al. 2019). Compared to other studies conducted in non–high-income economic countries, an investigation in India stated that 13% of the hospitals had basic IPC practices, 28% had intermediate and 59% had advanced IPC practices. In Sierra Leone and Pakistan, all hospitals had an inadequate and basic level of IPC (Kamara et al. 2022; Savul et al. 2020). It was also discovered that IPC scores were lower in hospitals in low-income and low-middle-income countries (Savul et al. 2020; Tomczyk et al. 2022).

Meanwhile, in this study, there was a difference in IPC level based on the type of hospitals, where the bigger ones have the better IPC level. Another study conducted in Indonesia revealed a strong correlation between IPCAF score and hospital classification. A similar report also discovered that referral hospitals have a better IPC level compared to others (Johannes et al. 2019). According to the 2019 Ministry of Health Regulations, hospitals in Indonesia are categorised based on the availability of specialist and subspecialist facilities and services. Therefore, hospitals of type B are better equipped than hospitals of types C and D. The Ministry of Health of the Republic of Indonesia has issued the most recent regulations regarding hospital classification in 2020, which categorise hospitals solely based on the number of beds; however, the current condition of the hospital reflects conditions according to the regulations in 2019, where type B hospitals have more complete facilities and beds than type C and D hospitals.

Based on the results, multimodal strategies for the implementation of IPC interventions (CC5) have the lowest minimum value among other components. The five elements for IPC multimodal strategies in a healthcare context include system change, training and education, monitoring and feedback, reminders, and communications as well as a culture of safety (World Health Organization 2009a). The multimodal IPC strategy can be applied on a large scale to successfully mitigate HAIs in the COVID-19 pandemic situation (Wee et al. 2021). The IPC team must determine the appropriate interventions to ensure the necessary infrastructure and the availability of sustainable care. It also needs to handle human interaction with the system, profession, principles, data, and methods in designing the optimal system according to needs, shortcomings, and human skills as well as accessibility. A recent study discovered that health workers have reduced new COVID-19 infections after the implementation of a multimodal strategy in the IPC programme (Karmarkar et al. 2021). Therefore, the current pandemic provides an opportunity to assess the effects of multimodal IPC bundles when used on a large scale (Wee et al. 2021). The implementation of the multimodal strategy includes system changes, education, training, monitoring and feedback, communication, and a safety culture recommended by WHO (World Health Organization 2009a).

In this study, it was discovered that there were low scores on the component HAIs surveillance (CC4). Furthermore, hospital-based surveillance systems, especially when connected to national surveillance networks, are associated with a reduction in HAI incidents (World Health Organization 2015). During the COVID-19 pandemic, the Ministry of Health of the Republic of Indonesia managed the systems based on the COVID-19 Prevention and Control Guideline (Ministry of Health of Indonesia 2020). The monitoring system allows the estimation of the local load and incidence of COVID-19. A previous investigation also discovered that the routine surveillance of IPC practices together with feedback is important to improve adherence to practices and reduce the burden of HAIs (Yinnon et al. 2012).

The IPC is one of the human resources team that will be responsible for monitoring the implementation of IPC programme as planned. The establishment of preparedness and response procedures in healthcare facilities for contagious disease emergencies including COVID-19 is also part of the IPC team’s task. The technical guidelines for the IPC programme that are updated according to the hospital capacity are essential to provide a strong design to promote good IPC practice. Previous research has revealed that IPC practice guidelines and procedures play an effective role in reducing HAIs, especially when applied in combination with the education and training of health workers (Loveday et al. 2014).

In the context of the pandemic, the IPC programme is one of the important things that must be enforced to carry out services in hospitals, especially during the COVID-19 pandemic. The WHO highly recommends that the IPC programme should exist in all healthcare facilities to reduce the incident of HAI (World Health Organization 2015). Infection prevention and control programmes that combine committed and trained personnel are effective in reducing HAI in hospitals (Mermel et al. 2013). During the COVID-19 pandemic, the success of implementation of IPC programme will provide benefits for hospitals and patients by reducing the incidence of COVID-19 cases.

However, during the pandemic, hospitals experienced shortages of PPE (Cohen, Van Der & Rodgers 2020). This condition also found COVID-19 in Indonesia (Sampe et al. 2021). The lack of PPE is one of the obstacles to adherence of IPC programme, which is often faced by hospitals in developing countries (Sharma et al. 2015). Moreover, evidence suggests that an enabling environment and adequate supply of basic facilities lead to increased compliance with IPC practices (Manojlovich et al. 2011). In addition, the lack of training for new staff members, communication barriers, and high workload are also barriers to adherence to the IPC programme (Barker et al. 2017). The lack of education coupled with the lack of resources and staff shortages that contributed during the COVID-19 pandemic had an impact on the implementation of the IPC programme.

Because this study involves only non-profit private hospitals, it cannot represent all public and private hospitals. There is also a tendency for differences in the IPC structures of these hospitals (Savul et al. 2020; Tomczyk et al. 2022). Furthermore, direct observations were not made due to the pandemic conditions in which the government and hospital management restrict people’s mobility as well as visits to COVID-19 referral hospitals.

## Conclusion

The results showed that most of the hospitals have adequate and intermedia levels of IPC; however, several changes are needed to improve the situation of IPC in private hospitals in Indonesia during the COVID-19 pandemic. The development and implementation of IPC guidelines to overcome the pandemic in line with hospital capacity must be carried out earlier. Finally, leadership support, multidisciplinary team involvement, and adequate training for infection prevention are needed to enhance the IPC programme (Weissenbach 2014). Hospital managers also need to provide an adequate supply to support the implementation of the programme.
